# Indication of air pollution based on the comparison of mercury and other elements in the faeces of marmots from the Western Carpathians and the Dzungarian Alatau

**DOI:** 10.1007/s11356-025-35902-w

**Published:** 2025-01-16

**Authors:** Martina Haas, Katarína Tomíková, Marián Janiga, Aibek Abduakassov, Zuzana Kompišová Ballová

**Affiliations:** 1 Institute of High Mountain Biology Žilina University, Tatranská Javorina 7, 05956 Tatranská Javorina, Slovak Republic; 2 Zhetysu University Named After I. Zhansugurov, Zhansugurov St., 187 A, 40009 Taldykorgan, Republic of Kazakhstan

**Keywords:** Mercury, Heavy metals, Chemical elements, Marmots, Alpine environment, Zhongar Alatau National Park, Western Tatras

## Abstract

The Dzungarian Alatau in Central Asia and the Western Carpathians in Central Europe are exposed to anthropogenic sources of pollution that are impacting high-altitude mountain systems through long-range transport of emissions. Based on analyses of the autumn faeces of two species of marmots (*Marmota baibacina* from the alpine habitats of Zhongar Alatau National Park, *Marmota marmota latirostris* from the alpine habitats of the Western Tatras), we determined the environmental load of mercury and other chemical elements. Our results show significantly higher levels of total mercury amounts (*p* < 0.0001) in faeces of marmots from the Western Tatras, Slovakia (mean = 0.066 µg/g dry weight; SD = 0.43), than in Zhongar Alatau National Park, Kazakhstan (mean = 0.034 µg/g dry weight; SD = 0.01), as well as sulphur and heavy metals (Ba, Mn, Mo, Zn, Cu, and Cr) that originate from anthropogenic activities. Other significant differences in levels of mineral nutrients (K, Cl, Ca, Fe) and Sr rather indicate differences in food sources reflecting environmental factors.

## Introduction

Current ecotoxicological studies focus on burden detection by determining the levels of toxic elements (e.g. Counotte et al. (2019); Pilarczyk et al. (2020); Tekeli et al. (2021)), including for heavy metals (e.g. Ali and Khan (2019); Jota Baptista et al. (2024)), in the tissues of animals at sites where there is likely to be obvious contamination of the environment by human activity, or the research is focused on animals that are sensitive to even small changes in the environment (e.g. sentinel and bioindicator animals; Amadi et al. 2022; Parmar et al. 2016). Non-destructive sampling techniques are widely adopted, given the ethical principles and legal standards associated with terminating live samples for the purposes of study. Therefore, faecal investigation has been proposed as a non-invasive technique to monitoring heavy metal exposure (Yasmeen et al. 2020).

Rodents are suitable indicators of environmental stress due to their habitat preference, and prevalence in both contaminated and uncontaminated sites, dietary composition, and increased exposure through ingestion and inhalation of contaminated soil (Ballová and Janiga 2018; Čadková et al. 2023; Ma et al. 2023). Marmots are long-lived burrowing rodents of high mountain areas, living from 13 to 15 years in the wild (Nowak 1991). Most marmot species occupy a harsh environment characterized by a short growing season and a long, cold season without food (Armitage 2014). The Tatra Mountains (Western Carpathians) are home to Tatra marmot (*Marmota marmota latirostris* Kratochvíl, 1961); it is a separate endemic subspecies of the alpine marmot (*M. marmota* Linnaeus, 1758) that lives only in the territory of the Western Tatras, Eastern Tatras, and Low Tatras Mountains (Ondruš et al. 2003). *M. marmota latirostris* occurs above continuous stands of dwarf pine, in the alpine vegetation zone. This subspecies inhabits habitats from 1500–1660 to 1900–2300 m a.s.l. (Ballová and Šibík 2015). Exceptionally, its burrow sets (colonies) are also found in places with extended dwarf mountain pine belts, in the subalpine vegetation zone. Tatra marmots hibernate for 7 months, usually from the second half of September or early October to the second half of April. One of the species of marmots occurring in Central Asia is the grey marmot, or Altai marmot (*Marmota baibacina* Kastschenko, 1899). It is one of the larger marmots in the genus *Marmota*. In south-eastern Kazakhstan and southwestern Siberia, their range extends to lower elevations, dry steppes, mountain steppes, and alpine meadows. They inhabit elevations from 150 to 4000 m a.s.l. (Řičánková et al. 2014; Wilson and Reeder 2005). Grey marmots hibernate from sometime between August and October for 7–8 months, depending on local conditions (Wilson and Reeder 2005). Marmot families of both species (Tatra marmot and grey marmot) consist of one dominant adult pair and their offspring of subordinate rank and of various ages (Arnold 1990; Bibikow 1996). Individuals from one marmot family inhabit a common home range area (Perrin et al. 1993). A home range of marmots is comprised of a core area, the main burrow system, and peripheral areas with shelter burrows (Blahout 1971; Perrin et al. 1993).

Soil-to-plant transfer of heavy metals is an entry point for toxic heavy metals and metalloids from the environment into food chains. It is the main pathway of exposure to soil metal contamination for organisms (Cui et al. 2004). Plants, as primary producers, bridge metal flows between abiotic and biotic components of ecosystems (Ali and Khan 2019). Marmot diet consists mainly of plants (grasses, berries, lichens, mosses), particularly lush herbs and soft parts of plants (shoots, leaves, heads, flowers), which are easily digestible and have a high nutritional value. Animal food seems to be negligible (Bibikow 1996). The presence of insect and animal remains in droppings has been documented mainly in spring and early summer (Ballová et al. 2016). Recent studies confirm a positive correlation of increased heavy metal content in both the environment and the plant (Palusińska et al. 2020). Similarly, airborne particulate matter content in polluted areas is significantly correlated with heavy metal content in plant leaves, suggesting a link with atmospheric deposition (De Temmerman et al. 2012; Fernández Espinosa and Rossini Oliva 2005). Food ecology is one of the main factors that accounts for the uptake of contaminants into an organism. In addition to this, the physiological state or developmental condition of the animal also plays an important role, which may alter the concentration of elements or, conversely, elevated concentration of contaminants may reduce an individual body condition (Ackerman et al. 2012, 2019; Peterson et al. 2021). Differences in MeHg exposure among sites can cause individuals of the same species that forage within the same habitat type to differ in MeHg bioaccumulation (Ackerman et al. 2008; Ackerman and Eagles-Smith 2010; Chételat et al. 2020).

Concentrations of pollutants increase with altitude; thus, alpine ecosystems become the main accumulation sites for heavy metals (Šoltés 1992). In terrestrial ecosystems, wildlife is mainly exposed to contaminants from the soil, and to a lesser extent to atmospheric or aquatic sources (Van den Brink et al. 2011). Soil contamination in high-altitude mountains is influenced by long-range air pollution (i.e. the transport of emission particles by global atmospheric flows). Densely populated and industrialized zones are the main sources of pollution, producing elevated levels of emissions. Long-range transport processes transport them to distant areas and mainly affect altitudes above the planetary boundary layer (1500 m a.s.l.) (Shotyk et al. 2002). Due to their spatial layout, high-altitude mountain ranges become barriers to air flow and the concentrations of heavy metals are significantly higher on the windward sides than on the leeward side of the mountains (Miśkowiec 2022).

Mercury is a widely discussed heavy metal known for its potent toxicity in the environment and status as a global pollutant (Driscoll et al. 2013; Kim et al. 2016; Ma et al. 2023). The amount of circulating mercury in nature has increased significantly over the last 200 years (Kalisińska 2019; Li et al. 2023). In alpine environments, melting glaciers (Zhang et al. 2012; Zheng 2015) and long-distance transport of pollution play a key role in maintaining atmospheric mercury deposition (Hylander and Goodsite 2006; Krabbenhoft and Sunderland 2013; Schroeder and Munthe 1998; Zhou et al. 2015). Terrestrial ecosystems can indicate long-term persistence of Hg introduced into the environment and the complexity of its transformation and circulation in nature (Kalisińska 2019). Atmospheric mercury is characterized by the variety of its chemical and physical forms. The organic form of mercury, methylmercury (Steinnes 1987), can be readily taken up by roots and subsequently stored in plants (Kabata-Pendias and Mukherjee 2007) and enters the food chain through herbivores. Undifferentiated forms of mercury (elemental, inorganic, and organic) are referred to as total mercury (T-Hg). Atmospheric Hg deposition can be broadly divided into wet and dry deposition. In addition to precipitation (e.g. rain, snow), non-precipitation forms such as clouds, fog, dew, and frost can be added to wet deposition of Hg (Stankwitz et al. 2012; Zhang et al. 2019). It is these types of non-precipitation Hg wet deposition that can play a crucial role in Hg contamination in alpine regions (Zhang et al. 2013a, b, 2019). Hg dry deposition is highly related to the underlying surfaces, including forest canopies, grasslands, wetlands, agricultural fields, deserts, background non-vegetated soils, and contaminated sites (Zhang et al. 2009). Dry deposition of Hg is significantly correlated with elevation (significantly higher dry deposition occurs at sites located at greater than 2000 m elevation), which may be a consequence of high concentrations of gaseous oxidized mercury (atmospheric Hg) and atmospheric turbulence (Huang and Gustin 2015).

By the end of the twentieth century, Asia was estimated to be the largest emitter of anthropogenic atmospheric pollutants, including trace elements (Pacyna and Pacyna 2001). Central Asian countries (e.g. Kazakhstan, Kyrgyzstan, Uzbekistan) are the main pollutant sources, due to large-scale mining industries and agricultural lands polluting regional ecosystems (Grigholm et al. 2016). It has been documented that the headwaters of the Ili River in the Dzungarian Alatau Mountains have elevated levels of Zn, Fe, Co, and Cr (Shen et al. 2022). The West Carpathians are one of the most polluted mountain ranges in Europe (Janiga et al. 2016). This region is exposed to air pollution transported from local sources and highly industrialized areas in southern Poland, the northern Czech Republic, and Germany (Degórska 2013; Krzan and Skawinski 1993). Environmental pollution through the fauna of the area was the focus of our previous studies (e.g. Ballová and Janiga (2018), Janiga et al. (2016)).

The aim of this study is to compare pollution by heavy metals, particularly total mercury (T-Hg), in two different mountain ranges in the Western Tatras (West Carpathians; Slovakia) and in Dzungarian Alatau (Kazakhastan). To compare the contamination of the area, we used a non-invasive method to investigate faeces of ecologically equivalent rodent species (*Marmota* sp.) from the alpine biotope, with the same food niches, during identical seasons. Excreted T-Hg levels in faeces correlate with dietary T-Hg intake (Grajewska et al. 2020), which ultimately provides a picture of the amounts of T-Hg in the environment that enter the food chain via plants as the main component of the marmot diet.

## Materials and methods

### Collection of samples

The study included faecal samples of two species of marmots—the grey marmot (*M. baibacina*) and the Tatra marmot (*M. marmota latirostris*). Collection of marmot faeces was conducted using a non-invasive method of collection directly from burrows in autumn (September–October 2022) at the beginning of hibernation to avoid disturbing the animals. The period before hibernation is the most suitable for collecting faeces samples, as marmots empty their gastrointestinal tract before hibernation, during which their metabolism slows down significantly (Ruf et al. 2022). Marmots usually defecate only in places within family territory, which can be of two types: open latrines in grazing territories and burrow latrines (blind hollows of maternal or winter burrows) (Ballová and Šibík 2015). Each latrine of both types (open or burrow) is utilized only by members of one family. Samples were randomly taken from both types of latrines. As it is difficult to distinguish droppings from different individuals, all samples from a single latrine burrow were labelled as a cumulative sample from the respective family. We always collected a few pieces of the freshest-looking droppings that were deposited on the surface of the faecal pile in a particular latrine burrow. We collected a total of 100 faecal cumulative samples coming from 100 latrine burrows (50 latrine burrows/cumulative samples from each country).

**Fig. 1:**
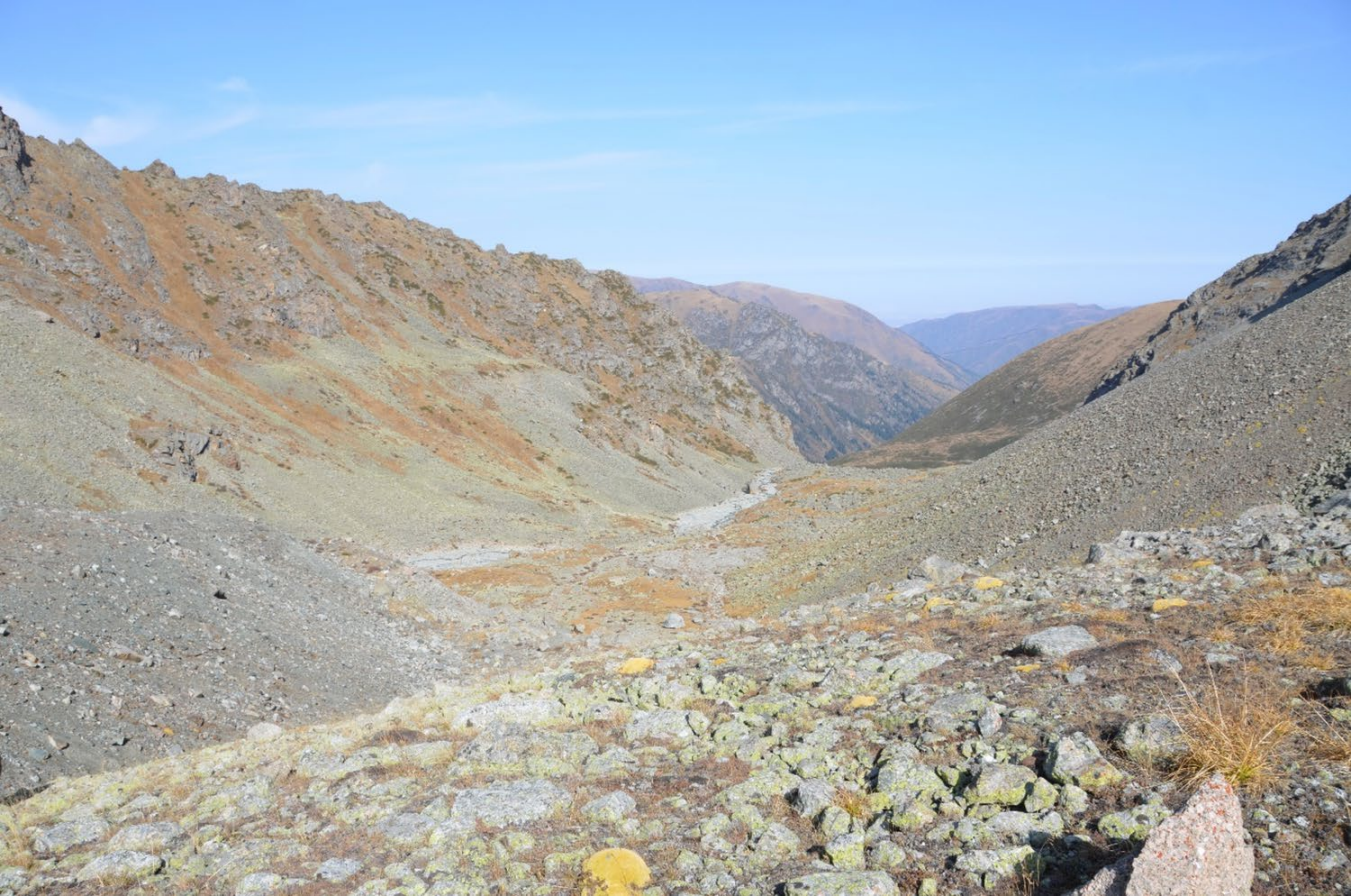
Habitat of grey marmot in the valley below the Shumsky Glacier in Zhongar Alatau (foto: M. Janiga)

In September 2022, colonies of the grey marmot (*M. baibacina*) were monitored and faecal cumulative samples were collected in Kazakhstan. The hibernating colony was located in a valley below the Shumsky Glacier in the Zhongar Alatau State National Nature Park (Sarkand District; 45.096266 N; 80.219657 E; 3250 m a.s.l.). The park sits on the northern slope of the Dzungarian Alatau. Dzungarian Alatau, a range between the Tian Shan to the south and the Altai Mountains to the north, is one of the largest and structurally complex mountain systems on the border of Kazakhstan and China (Šlégl 2001). The mountain range is located between the Ili River and the Alakol Lake. The territory is characterized by an extremely continental to arid climate (Merzlyakova 2003). The climate on high mountain slopes is mild, with cold winters (average air temperature in January is − 8.5 °C) (Andasbayev et al. 2013) and cool summers (average air temperature in July is 19 °C) (Beisenova 2014). From an elevation of 2750 m a.s.l., alpine vegetation is represented by grasslands and low shrub forms of willow (Fig. 1).

**Fig. 2:**
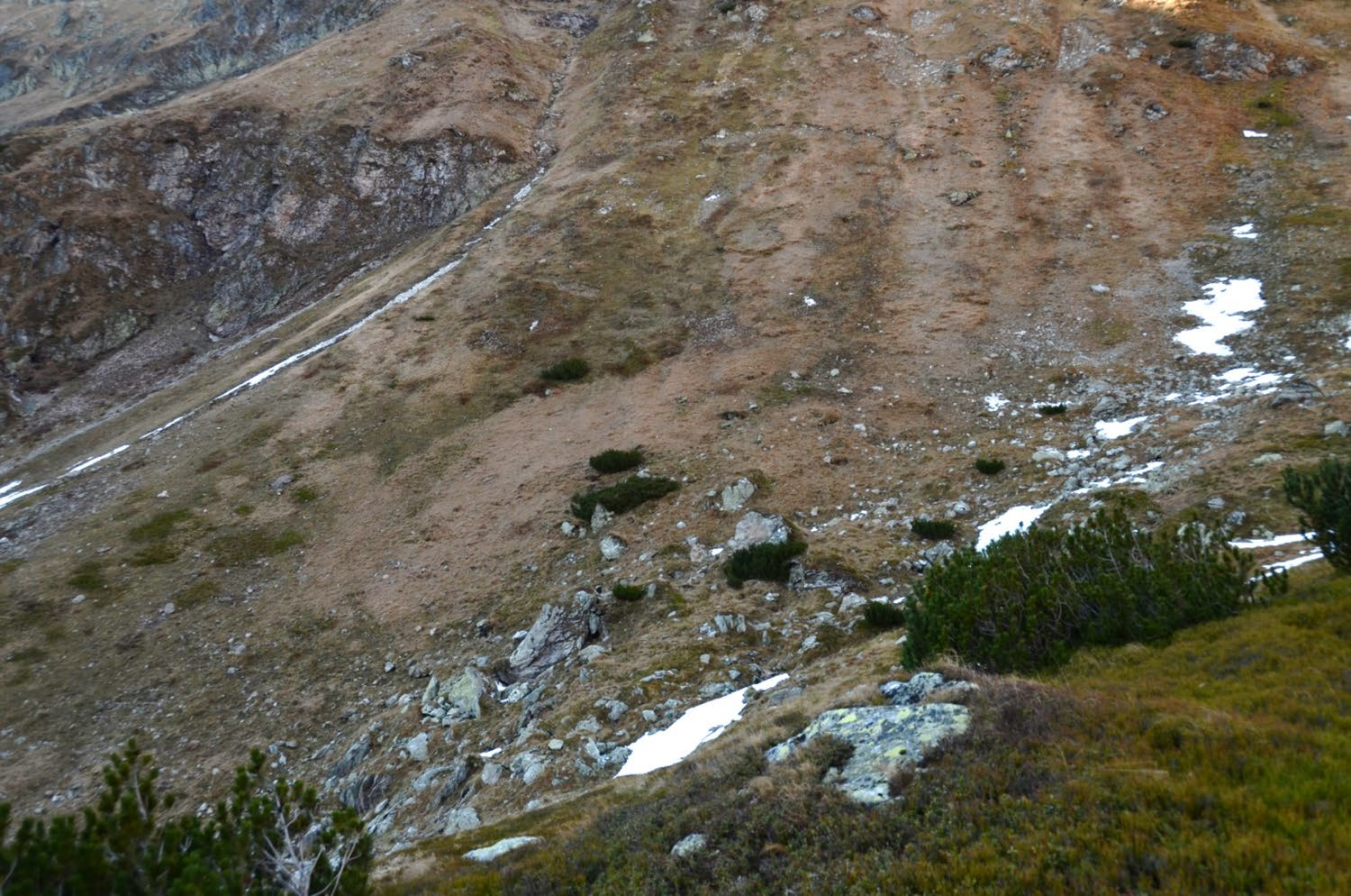
Habitat of marmot colony in Račkova valley, Western Tatras (foto: M. Haas)

In October 2022, marmot colonies were monitored and faecal cumulative samples were collected in the Western Tatras, Slovakia (Fig. 2). As colonies are smaller in this area than in Zhongar Alatau, we focused on the adjacent valleys: Jamnická valley: 49.20357 N, 20.01151 E, 1738 m a.s.l.; Račkova valley: 49.19712 N, 19.8127 E, 1703 m a.s.l.; Bystrá valley: 49.17942 N, 19.83602 E, 1859 m a.s.l.; and Kobylia valley: 49.20433 N, 20.01151 E, 1711 m a.s.l. The Western Tatras are one of two geomorphological subdivisions of the Tatra Mountains (province of the Western Carpathians). The territory of the Western Tatras belongs to the Tatra National Park and has a cold mountain climate. The average air temperature in July reaches 6 °C and average January temperatures reach − 9 °C. The alpine zone extends to an altitude of about 2300 m a.s.l. It consists of the original, primary alpine meadows that extend above the dwarf pine (*Pinus mugo*) belt.

Despite differing elevation, the biotopes in both locations have the characteristics of an alpine ecosystem, which is defined as areas with plant communities occurring above tree line elevation, by extreme winter temperatures, lower availability of nutrients and water, low ambient temperature, shorter growing seasons, and higher UV radiation compared to lowland ecosystems (Barbour and Billings 2000; Bowman and Seastedt 2001; Körner 1999).

### Analysis of mercury (T-Hg) and other elements

One hundred cumulative samples (50 from each country) of approximately the same size were formed in the laboratory. The size of each cumulative sample was adjusted so that each sample contained approximately the same amount of faeces from one burrow (“latrine burrow”) belonging to a particular family. All samples were air dried at room temperature in the laboratory for 5 days. The top layer was then scalpel scraped to remove any potential contamination from extraneous materials such as rocks, soil or vegetation.

After scraping, samples were homogenized (by hand in a laboratory mortar) and analyzed by X-ray fluorescence using a handheld ED-XRF spectrometer DELTA (Innov-X Systems, USA). The thickness of the measured sample in the plastic container (part of the XRF accessory) was 0.5 cm after squeezing, as standard. The sample was measured for 240 s in three repetitions, from which the average was calculated. All samples were measured in default mode. The detection limits were determined continuously for each measurement and for each element by software using the Compton normalization method. Only elements (S, Cl, K, Ca, Cr, Mn, Fe, Cu, Zn, Rb, Sr, Mo, Ba) measured above the detection limit were included in statistical analyses. Standard reference material INCT-PVTL-6 Virginia tobacco leaves (ICHTJ, Poland) was used to standardize the measurement method. The calibration factor was calculated for each element as the ratio of the average values of three measurements of the reference material and the certified values. This calibration factor was used in the analysis of faecal samples. Control measurements of standard reference materials agreed well with the certified values within the uncertainty of relative standard deviation (RSD) limits better than < 10% (RSD).

Total mercury levels in faecal samples were measured by thermal decomposition, amalgamated with a direct mercury analyzer (DMA-80 Dual Cell, Milestone, Italy). Each sample was weighed using a precision scale Kern 770 (Kern&Sohn, Germany) in a metal boat (DMA 8142). The temperatures in the method were set to the following values: combustion temperature 750 °C; catalyst temperature 615 °C; cuvette temperature 125 °C. Mercury concentration was calculated automatically as the measurements were calibrated to each sample’s weight and the levels of mercury were determined on a dry weight basis. A sample of the certified reference material INCT-PVTL-6 Virginia tobacco leaves (ICHTI, Poland) was used to check the quality of the measurements and to validate the measurement method. The limit of detection (LOD) and the limit of quantification (LOQ) were established by ten independent analyses of the blank (empty nickel boat). The following equations were used to calculate LOD and LOQ: LOD = 3 * *s* and LOQ = 10 * *s*, where *s* = average standard deviation, calculated from 10 independent determinations of the blank. LOD and LOQ were determined as 0.003 µg/g and 0.0092 µg/g, respectively. The method represented a high average percentage recovery (> 95%) in the standard reference material matrix and a relative standard deviation (RSD) less than or equal to 5%.

### Statistical analysis

Element values in marmot droppings obtained from the XRF analysis and from the direct mercury analyzer were statistically evaluated using Statistica ver. 12 (StatSoft, Inc.). According to the Shapiro–Wilk test, the data was not distributed normally. To compare T-Hg levels between sample groups from Slovakia and Kazakhstan, we used the non-parametric Kruskal–Wallis H test. Principal component analysis (PCA) was used to assess the relationships between the elements accumulated in faeces. The use of PCA is an appropriate method for capturing interactions between variables because this multivariate method reduces the dimensionality of the data and identifies patterns in the data while creating new variables (factors/principal components) whose effect can be further tested. This technique is widely used in many environmental studies to discover the possibility of synergistic interactions between different pollutants or other elements. In some cases, the co-accumulation of multiple elements, even at relatively low concentrations, can exert a more pronounced impact through a synergistic effect. The correlation matrix was used as a basis for further analysis, because it normalizes the data when individual variables (element levels) are measured on different scales (Jolliffe 2002). Factor-variable correlations were then calculated. These are correlations of the original variables (elements) with the newly created variable (factor). In other words, a principal component is a linear combination of the variables that are most highly correlated with it. We considered strong correlations to be those with a value between 0.5 and 1.0 (Table 1). The factors were further tested using the Kruskal–Wallis H test (95% confidence level; *p* < 0.05) to determine the difference in the pattern of element accumulation between the compared countries. Further analyses were performed using component scores. Component scores are quantitative values of individual samples in a phenomenon. In the plots (Fig. 4a–c), the numbers on the y-axis are the values of the principal component scores, with higher numbers indicating higher levels of element accumulation. Negative values (scores) indicate a negative relationship of the variable with the factor, and a positive direction of the factor indicates a decreasing strength of the effect of variables with a negative value, which in our case represent the levels of the elements. A negative score of a variable (= negative relationship with the factor) with a simultaneous negative direction of the factor means the opposite, i.e. a positive manifestation of the variable.

**Table 1:** Factor-variable correlations (factor loadings) with percentage of variance in principal component analysis of accumulation of chemical elements in marmot faeces

Elements	Factor 1	Factor 2	Factor 3	Factor 4
S	** − 0.852**	0.195	0.248	0.006
Cl	0.203	**0.742**	0.440	0.041
K	0.234	**0.800**	**0.509**	0.000
Ca	0.049	** − 0.670**	**0.571**	− 0.220
Cr	** − 0.598**	0.206	− 0.244	− 0.485
Mn	** − 0.775**	0.181	0.142	0.315
Fe	− 0.167	0.158	− 0.125	** − 0.828**
Cu	** − 0.600**	0.222	0.104	0.312
Zn	** − 0.620**	− 0.252	0.470	0.095
Rb	0.477	**0.578**	0.329	− 0.315
Sr	0.156	** − 0.665**	**0.589**	− 0.144
Mo	** − 0.678**	0.195	− 0.223	0.017
Ba	** − 0.845**	− 0.106	0.152	− 0.299
% Total variance	**30.6**	**20.9**	**13.5**	**10.7**

## Results

### Amount of mercury in the faeces

**Fig. 3:**
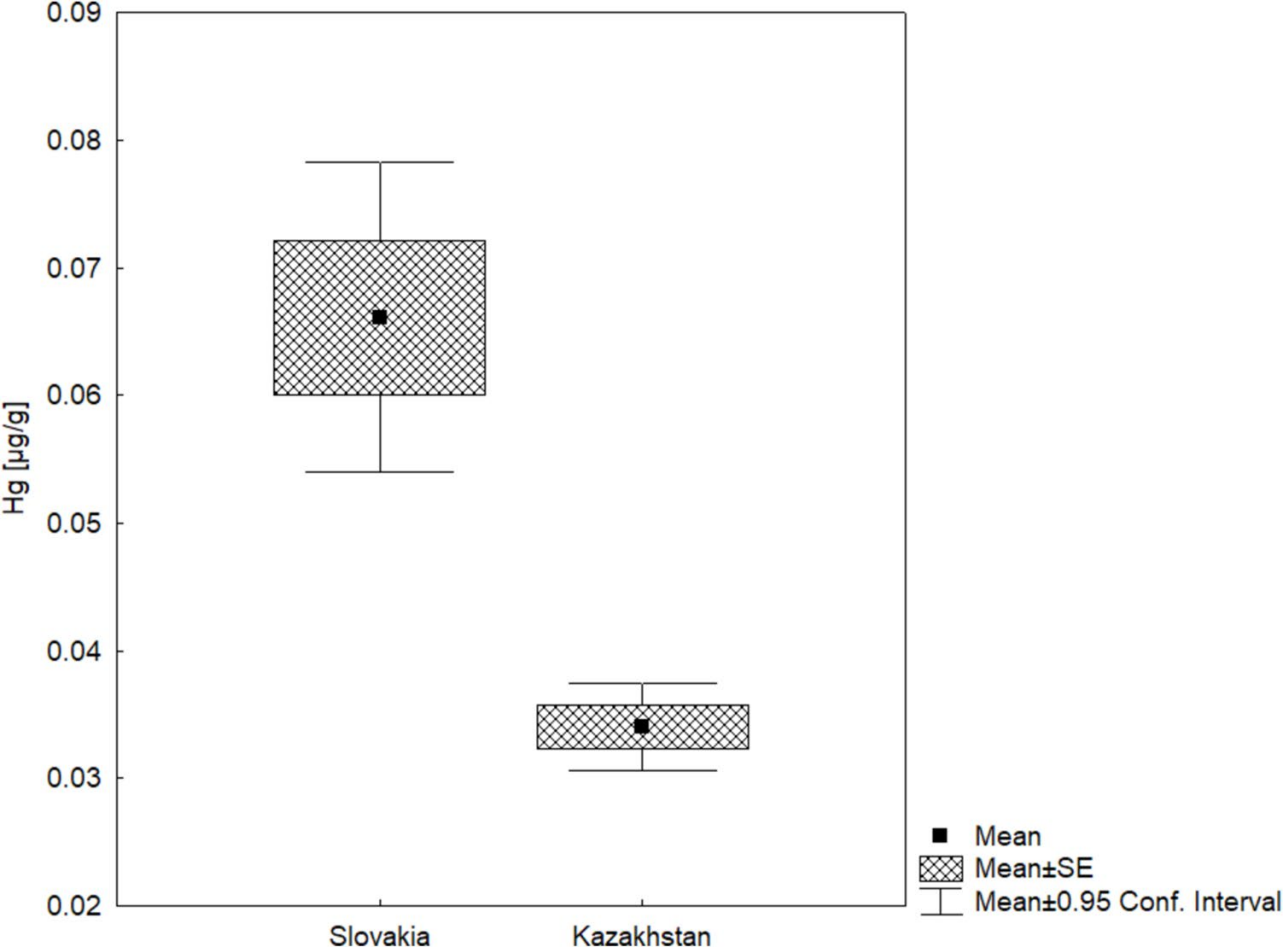
Comparison of Hg content (µg/g dry weight) in the faeces of marmots from Slovakia and Kazakhstan KW-H test (1, 100) = 16.4314; *p* < 0.0001

In a comparison of samples from both countries (Fig. 3), the amounts of T-Hg (µg/g dry weight) were significantly higher in faeces of marmots from Slovakia (mean = 0.066; SD = 0.43), than in Kazakhstan (mean = 0.034; SD = 0.01). Mercury levels in faeces from Slovakia were also more variable.

### Accumulation of other chemical elements in faeces

The first four factors explained the most variability in the data and accounted for more than 10% of the variance (total more than 75% of the variance). Factor-variable correlations (factor loadings) of the four most important principal components based on correlations are shown in Table 1.

**Fig. 4:**
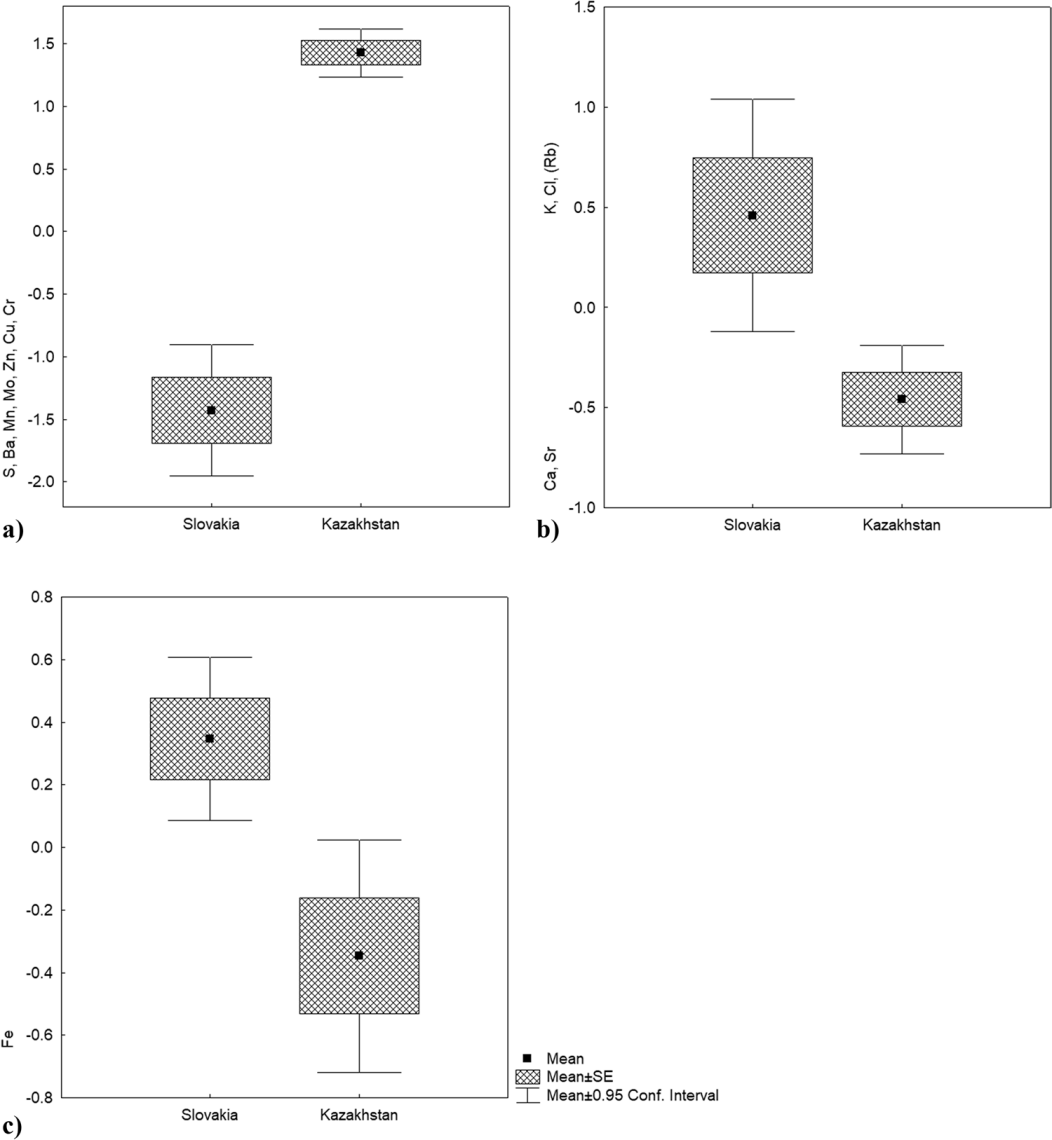
The most significant phenomena of element accumulation in marmot faeces by comparison of PCA scores on individual components between Slovakia and Kazakhstan. **a** Significantly higher mutual accumulation of S, Ba, Mn, Mo, Zn, Cu, and Cr in marmot faeces from Slovakia than in the faeces from Kazakhstan (KW-H (1;100) = 63.3992; *p* < 0.0001). **b** Significantly higher values of K, Cl, and partly Rb and lower values of Ca and Sr detected in the faeces of marmots from Slovakia than in the faeces from Kazakhstan (KW-H (1;100) = 13.552; *p* < 0.001). **c** Significantly higher Fe content in samples of marmot faeces from Kazakhstan than in samples from Slovakia (KW-H (1;100) = 16.4314; *p* < 0.0001)

The most significant phenomenon occurred in the mutual accumulation of the elements S, Cr, Mn, Cu, Zn, Mo, and Ba (factor 1). Such accumulation in faeces of marmots was significantly higher in Slovakia than in Kazakhstan and the accumulation of elements in faeces varies more in Slovakian samples than those from Kazakhstan (Fig. 4a). Another significant factor is the bipolar vector and represents an inverse mutual relationship between the K and Cl (Rb) group and the Ca and Sr group. An increase in the levels of the elements K and Cl leads to a decrease in the levels of the elements Ca and Sr and vice versa. This factor differs in the compared areas, the elements K and Cl (Rb) contribute significantly to the strength of the factor in Slovakia, i.e. their content in faeces is higher, while the levels of Ca and Sr were significantly lower than in faeces from Kazakhstan (Fig. 4b). Factor 3 accounts for differences in the accumulation of the elements Sr, Ca, and K and is independent of factor 2. Although the values of principal component scores tend to be lower in Kazakhstan than in Slovakia, the marmot faeces did not differ significantly in this phenomenon (KW-H (1;100) = 2.0561; n.s.). Factor 4 is a unipolar factor that reveals the amount of Fe in the faeces of marmots between the compared countries. Fe levels in faeces were significantly higher in samples from Kazakhstan (Fig. 4c). The level of Fe in faeces is clearly dependent on the environment.

## Discussion

Significant differences in the amount of mercury we detected in the faeces of marmots prove its higher levels in the environment of the Western Tatras. We can confirm that the West Carpathians are more burdened by pollution originating from anthropogenic activities than Dzungarian Alatau. This area is also more polluted than Tian Shan (Ballová and Janiga 2018) and most of European mountains (Janiga et al. 2016). Substances from the soil, water, and plants enter the digestive system with the animals’ food (Tölgyessy et al. 1989). Patterns of accumulation of mercury and other chemical elements are related to the diet of animals in different habitats, as the type of food may exhibit habitat-specific availability (Vermeulen et al. 2009). Although an analysis of food sources was not part of our study, we hypothesize that food, as well as the ecology of the study species (e.g. possible aspiration of soil particles during burrow excavation), is a major source of mercury entry into the body of marmots. Toxic substances are accumulated in the body’s organs or excreted. Accumulation of metals is much easier than their elimination. Thus, once metals are accumulated in body tissues, it is difficult to eliminate them and this is especially true for non-essential metals (Kalay and Canli 2000). However, it was found that 90% of methylmercury ingested in food and absorbed by the respiratory tract is excreted through faeces (Ye et al. 2016).

Other results of this study, including the analysis of metallic elements in faeces, show a significant relationship of a group of elements (S, Ba, Mn, Mo, and slightly less contributing Zn, Cu, Cr) with the area of the Western Tatras (factor 1). These elements, in addition to sulphur, are also considered heavy metals (Orlov et al. 2002). Some of these elements (Mn, Cu, Zn) are essential heavy metals, and although they are part of biochemical and physiological functions, they can also be potentially toxic if present in excess (Sauliutė and Svecevičius 2015). Concentrations of Mn, Cu, and Cr present in topsoil in the Tatra Mountains reach low to average values, according to Tóth et al. (2016). Wang et al. (2021) reported that metal particles (Cu, Zn, and Mo) in urban dust are mainly from anthropogenic sources. Mn and Ba are slightly influenced by human activities, and Cr comes from natural sources. Increased mobility of these elements in the environment through anthropogenic activities is also reported by Kara et al. (2014). A higher concentration of Cr can also be caused by coal mining (Shen et al. 2022). The higher concentration of sulphur in mountain soil and plants is mainly a consequence of air pollution with sulphur dioxide (Kandziora et al. 2009). Based on these findings, we can conclude that factor 1 represents the load of heavy metals and sulphur in the area, which originates from anthropogenic sources, and is also related to the long-range transport of pollutants in the environment, deposition in the topsoil, and entry into the food chain through plants. Zhongar Alatau State National Nature Park is located in a belt of mountain fruit woods, in the south-eastern territory of Kazakhstan on the China-Kazakhstan border. Atmospheric flow can bring in emissions from the main industrial centres, which are located in regions of Central and East Kazakhstan. In this region, approximately 3–4 million tonnes of polluting chemicals are released into the air or deposited on the land surface annually (Saparov 2014). Despite the fact that there are no major industrial centres located in close proximity, according to Cherednichenko et al. (2021), the territory represents a mountainous area where the values of the maximum permissible concentration of heavy metals (Pb, Cd, Cu, and As) in the soil reach 5–50 µg/L. Similarly, elevated concentrations of heavy metals (Pb, Cd, Cu, Zn, Ni, Fe, Co, Mn, Sr, Cr) on the northern slopes of the Dzungarian Alatau exceeded the values of the maximum concentration limit (Andasbayev et al. 2013). The Tatra National Park is situated in North Central Slovakia in the Tatra Mountains. The pollution in the Tatras is caused by long-distance transport of emissions from industrial and mining zones located mainly in north-eastern Czech Republic and southern Poland (Degórska 2013; Krzan and Skawinski 1993). Between 2005 and 2020, emissions of heavy metals (Cd, Pb, Hg) decreased by at least 10% in most EU Member States, with Germany, Italy, and Poland remaining the largest emitters, accounting for about half of the EU total for all three heavy metals (EEA 2020). Pollutants are deposited through long-distance transport, where air flows deposit them in high mountain ranges. The Tatras represent a barrier for atmospheric pollutants from the north west part of Europe (Ballová and Janiga 2018) and probably because of this, the impact of pollution from anthropogenic activities is greater there than in Dzungarian Alatau.

The second factor reflected the relationship between excreted elements (Ca, Sr, K, and Cl). This factor is positively expressed in Slovakia, i.e. the increase in the variables K and Cl (Rb) and the decrease in the variables Ca and Sr are more pronounced in Slovakia. We conclude that this factor is not related to environmental pollution, but quality of food sources. Potassium and chlorine are important for soil fertility, similar to other necessary elements in plant nutrition and growth (e.g. Geilfus (2019); Srivastava et al. (2020)). Calcium is considered a significant plant and animal growth regulator. Thus, plants are the main source of potassium and calcium intake in animal diets (Reid and Horvath 1980). Plants receive nutrients most often in the form of soil solution, and the intake of nutrients is influenced by both internal (genetic) factors and external factors (conditions of the habitat, soil, climate) (Tůma et al. 2004). We assume that environmental factors influence the representation of nutrients in plant food the most. Soil nutrients and foliar macronutrient concentrations of nitrogen, magnesium, potassium, and foliar phosphorus decrease significantly with elevation (Drollinger et al. 2017). The grey marmot colony in Zhongar Alatau is situated at a higher elevation, which can negatively affect the amount of potassium in food. Another crucial factor is climate; in Zhongar Alatau, a continental climate with warmer summers and lower precipitation prevails, unlike the Western Carpathians. In dry areas, grasslands have fewer minerals (Preston and John 1985). The length of the active photosynthetic phase of the plants also directly depends on the humidity and amount of water in the environment. According to the study by Krendželák et al. (2018), the amount of potassium and chlorine in *Juncus trifidus* from the Tatra Mountains is the highest in the summer months in the green parts of the plants, and the amount of calcium is the highest in the dry plant, in autumn. Potassium and calcium intake appear to be ambivalent (Rafferty and Heaney 2008), which is consistent with our conclusion.

The last significant factor (factor 4) is related to iron content in faeces. Fe is significantly more excreted in the faeces by marmots from Zhongar Alatau. Iron is contained in minerals and rocks and is released into the soil through weathering. The main source of iron in soils for use by plants comes from secondary oxides absorbed or precipitated onto soil mineral particles and iron-organic matter complexes (Jones 2020). Plant roots absorb iron from the soil solution most readily as ferrous, but in some cases, also as ferric ions (Kobayashi and Nishizawa 2012). Among plant organs, it is the roots that contain the highest concentrations of iron (Krendželák et al. 2018). In iron-poor soils, iron content is also low in plants. There is a strong correlation between available iron levels in soil and the occurrence of iron deficiency anaemia, which is caused by dietary iron deficiency (Brown et al. 1996). Generally, high pH reduces iron solubility and therefore, iron deficiency is found in calcareous soils (Patra et al. 2021). The soils of the Western Tatras are rated as acidic due to their geological substrate, but also due to anthropogenic processes (Bowman et al. 2008). However, there are iron ores in the Dzungarian Alatau and the soils are rich in iron deposits, which may influence the availability of dietary iron and explain its higher levels in faeces from the Dzungarian Alatau.

## Conclusions

Based on the comparison of heavy metal concentrations and other chemical elements present in marmot faeces, we concluded that the Western Carpathians are still one of the most vulnerable sites in terms of anthropogenic pollutant load, despite current trends in emission reduction and the transition to a green economy. We found that the Western Tatras are more polluted with elements (Hg, S, Ba, Mn, Mo, Zn, Cu and Cr) that originate from anthropogenic activities than Zhongar Alatau. Prevailing north-western winds and the long-lasting legacy of mining and smelting in the area of the Western Carpathians cause this pollution. Pollution by heavy metals and potentially toxic elements may also be a consequence of the longer history of industrialization in Western Europe, as well as the relatively shorter distances between the sources of pollutants from European countries to the sampling sites in the Western Tatras. Conversely, the high mountain areas in the Dzungarian Alatau, where the samples were collected, are much further away from the industrialized centres, as the valleys in this mountain range are more extensive than the Carpathian valleys. Other significant differences in the levels of the elements analyzed (K, Cl, Ca, Sr, Fe) are related to food sources (i.e. plant food). Environmental factors such as soil composition, plant species composition, rainfall, and climate are most responsible for the differences in the accumulation of these elements.

## Data Availability

The datasets used and analyzed during the current study are available from the corresponding author on reasonable request.

## Declarations

### Author consents of participation and publication

All authors read and approved the final manuscript. All of them have agreed on submitting the manuscript to Environmental Science and Pollution Research.
